# Extended Object Tracking with Embedded Classification

**DOI:** 10.3390/s22062134

**Published:** 2022-03-09

**Authors:** Wen Cao, Qiwei Li

**Affiliations:** 1 School of Electronics and Control Engineering, Chang’an University, Xi’an 710064, China; 2 School of Electronics and Information, Northwestern Polytechnical University, Xi’an 710129, China; liqiwei161@163.com

**Keywords:** extended object tracking, soft classification, hard classification, random matrix, sequential probability ratio test (SPRT)

## Abstract

This paper proposes a novel extended object tracking (EOT) approach with embedded classification. Traditionally, for extended objects, only tracking is addressed without considering classification. This has serious defects: On the one hand, some practical EOT problems require classification as an embedded subproblem; on the other hand, with the assistance of classification, the tracking performance can be improved. Therefore, we propose a systematic EOT method with embedded classification, which is desired to satisfy the practical demands and also enjoys superior tracking performance. Specifically, we first formulate the EOT problem with embedded classification by kinematic models and attribute models. Then, we explore a random-matrix-based, multiple model EOT method with embedded classification. Two strategies are creatively provided in which soft classification and hard classification are embedded, respectively. Especially for the EOT with hard classification, a sequential probability ratio-test-based classification scheme is explored due to its nice properties and adaptability to our problem. In both methods, classification assist tracking is used. The simulation results demonstrate the superiority of the proposed EOT method with embedded classification, which can not only satisfy the practical requirements for classification but can also improve the tracking performance by utilizing the assistant of classification.

## 1. Introduction

Target tracking is a critical problem in land-based airborne surveillance systems, with abundant results [1,2,3,4,5,6,7,8,9]. Traditionally, due to limited sensor resolution and large sensor error relative to the target size, a target is usually considered as a point target, and only the kinematic state (position, velocity, acceleration, etc.) is considered. With the development of sensor resolution, multiple measurements from one target are available, and thus, considering the object extension is possible. Therefore, extended object tracking (EOT) arises, in which both kinematic state estimation and object extension (i.e., size, shape, and orientation) are estimated [10,11,12,13,14,15,16,17].

Several approaches have been proposed for extended object tracking, e.g., multiple hypothesis tracking [18], probability hypothesis density filters [15], hyper-surface model [16], the sequential Monte Carlo method, and a method based on support functions and extended Gaussian images [13]. An approach of using a random matrix initiated by Koch [10] and developed by [11,12] is promising. This approach treats the extended object as one object, and its final form is simple. Specifically, it estimates both the kinematic state represented by a random vector and the extension represented by a symmetric positive definite (SPD) random matrix. In [12], two new random-matrix-based models describing evolution and observation distortion of the object extension were proposed to estimate the kinematic state and extension jointly with promising simplicity and effectiveness.

In the literature on extended object tracking approaches, only object tracking is handled [10,11,12]. This is natural and direct since the final goal of EOT is to obtain an accurate estimate about the object’s state and extension. For targets in reality, however, in addition to the state information, it also has a class label (e.g., the target maybe a bomber, a fighter, a commercial jet, etc.), and determining which class the object belongs to is essentially a classification problem.

However, in practice, some problems about extended object require only tracking, while some others require classification as a subproblem in addition to tracking. For the former, although object classification is not required, the object class information helps improve the tracking performance. Specifically, most tracking algorithms are based on motion models while classification facilitates tracking via selecting appropriate class-dependent kinematic models. This helps improve the tracking performance in an indirect way. However, most traditional EOT methods do not consider this important classification information, which leads to limited tracking performance. Alternatively, some studies may realize that classification benefits tracking but do not know how to utilize the classification information to assist object tracking. The main reason is that data used for classification usually have different characteristics from that for tracking, which may not be used for tracking directly. For example, some sensors provide indicative information, which indicates the target identity but cannot provide target state information, and thus it cannot be used for tracking directly. For EOT problems requiring classification as a subproblem, classification plays double roles: On the one hand, it can facilitate tracking as analyzed above; on the other hand, it is a goal that must be achieved.

In view of the above, EOT with embedded classification is critical and is urgently required to be solved. This is expected to not only satisfy the practical requirements but also achieve superior tracking performance due to the assistance of classification. Traditionally, tracking and classification are usually treated separately and are solved using their respective data and techniques. Object tracking are usually based on kinematic (e.g., radar and sonar) measurements, which can provide state information directly, e.g., position, velocity, etc. Object classification is usually handled using identity or attribute data from high-resolution radar, electronic system measurement (ESM), passive infrared, electrometrical imaging sensors, or radar cross-section (RCS) [4,19,20], which can provide critical class information but do not contain the kinematic state information and cannot be used for tracking directly. By introducing classification into EOT, more heterogeneous sensor data can be utilized for tracking, which are used only for classification before.

Therefore, we consider EOT with embedded classification in this paper. We first formulate the extended object tracking with an embedded classification problem explicitly by modeling. For target state evolution, a hybrid dynamic model describing both the evolution of kinematic state and extension is presented. For attribute evolution, we adopt the ESM data, and the corresponding feature evolution mode and ESM measurement model are presented. Considering this, in reality, there are different demands for classification in EOT. Specifically, some may not care about the target class but only the tracking performance. In some other cases, however, although the ultimate goal is tracking and we devote time to improving the tracking performance, the explicit class label is also required as a subproblem. Therefore, in the former, we no not need to determine the hard object class, and the only goal is to improve tracking; in the latter, we should also make a hard decision on class label in addition to tracking.

To satisfy the above demands, we provide two strategies for EOT with embedded classification, that is, soft-classification-aided tracking and hard-classification-aided tracking. Specifically, for tracking, the random-matrix-based EOT method is adopted with a multiple model approach, and for classification, both kinematic data and attribute data are utilized. Based on these, in EOT with soft classification, at every time step, the tracking results and the probability of each object class (i.e., soft classification) are output. In EOT with hard classification, we propose to adopt the well-known SPRT decision strategy due to its nice properties, and the estimation is output according to whether the hard classification is made. Note that in both EOT methods, classification assists tracking. Simulation results demonstrate the effectiveness of the proposed random-matrix-based EOT method with embedded classification.

The novelties of this paper are as follows.

(1) We formulate the extended object tracking with embedded classification problem while most existing literatures for extended object only consider tracking. Specifically, a kinematic state and extension dynamic model, a state measurement model, an attribute evolution model, and an attribute measurement model are all presented for modeling.
(2) We propose the random-matrix-based EOT method with embedded soft classification. This is for practical problems requiring object tracking only. Multi-sensor data are utilized for helping the soft decision, which finally assists object tracking.
(3) We propose the random-matrix-based EOT method with embedded hard classification. This is for practical problems requiring both tracking and explicit hard classification as a subproblem. The SPRT decision strategy is adopted for its nice properties and also adaptivity to our problem.
(4) Simulation results demonstrate the effectiveness of the proposed two EOT strategies with embedded classification. The assist of classification to tracking is verified, and the indirect assistance of attribute data to tracking is also verified.

This paper is organized as follows. Section II formulates the EOT with embedded classification problems by modeling the kinematic state, extension, and attributes. Section III, as the main part of the paper, proposes the novel EOT method with embedded classification. The random-matrix-based multiple model EOT methods with embedded soft classification and hard classification are proposed, respectively. Section IV presents the simulation results. Section V concludes the paper.

## 2. Problem Formulation

Consider that there is only one extended object with two possible classes (e.g., a fighter or an airline), which have different maneuverabilities. For an extended object, maneuverability is reflected in both the kinematic state (change in position, velocity, etc.) and extension (change in size, shape, orientation due to, e.g., rotation). In addition, different classes of objects may have different feature attributes, which contain the object class information. Our goal is to track the extended object and also classify the object as a subproblem according to the practical demands using all available data. Practically, for an extended object, some require only tracking while others require additional classification of the object class label.

To handle this problem, we first need to model it using state (kinematic state and extension) models and attribute models. Specifically, radar provides kinematic data containing the target state information, while an attribute sensor provides the target class information. This paper adopts the electronic support measure (ESM) for feature attributes.

### 2.1. State Model

#### 2.1.1. State Dynamic Model

For extended object tracking, reference [10] proposed the random-matrix-based EOT method, which has superior performance and a simple implementation. Based on this, we also develop it by using the Planar Constant-Turn (PCT) model [21,22]. Specifically, with the state $x=[x,\dot{x},\ddot{x},y,\dot{y},\ddot{y},z,\dot{z},\ddot{z}]$, the kinematic evolution motion for EOT is:

(1) $$x_{k+1}=\mathrm{diag}\left[F\left(\omega \right),F\left(\omega \right),F\left(\omega \right)\right]x_{k}+w_{k}$$

where F(ω) and the measurement noise $w_{k}$ are omitted with details shown in [22].

The extension dynamic model is given by [12,23]:

(2) $$p\left[X_{k}|X_{k-1}\right]=W\left(X_{k};\delta_{k},A_{k}X_{k-1}{\left(A_{k}\right)}^{\prime }\right)$$

where W(Y;a,C) is the density of the Wishart distribution of the SPD random matrix $Y\in R^{d\times d}$, $\delta_{k}>d-1$ is the corresponding degrees of freedom, and $A_{k}\in R^{d\times d}$ describes the specific evolution mode.

**Remark** **1.** *With the above models (1) and (2), the dynamics of the kinematic state and extension are both described. Furthermore, they have many advantages, e.g., Equation (1) can describe the target centroid turn, and the coupling between the centroid maneuver and the extension rotation is also explicitly described [22].*

#### 2.1.2. Measurement Model

In practical EOT problems, we can obtain multiple measurements from one extended object. Assume that the radar measurements are observed positions of object scattering centers, and denote by $Z_{k}={\left\{z_{k}^{r}\right\}}_{r=1}^{n_{k}}$ a set of $n_{k}$ vector-valued position measurements at time *k*. The measurement model is given by [12]

(3) $$z_{k}^{r}={\tilde{H}}_{k}x_{k}+v_{k}^{r},\;v_{k}^{r}∼N\left(0,B_{k}X_{k}{\left(B_{k}\right)}^{\prime }\right)$$

Here, the measurement matrix ${\tilde{H}}_{k}=H_{k}⊗I_{d}$, where *d* is the dimension of space for EOT, $H_{k}=[100]$ is the measurement matrix in the one-dimensional space, and $v_{k}^{r}$ is independent Gaussian white noise [12,23].

### 2.2. Feature Model

Among the various multi-sensor data, an ESM sensor is a passive directional receiver which scans the frequency range of interest to intercept emitted electromagnetic signals from targets and identifies the likely source emitters [5]. Therefore, ESM contains target class information, and thus can be applied for classification naturally. This paper adopts the typical ESM as the feature attribute with the assumptions: Emitters may be on or off (attribute evolution process); detected emitters may be confused with other emitters (measurement process).

Specifically, suppose there are two possible classes, $c_{1}$ and $c_{2}$, where $c_{1}$ has emitter $E^{1}$ and $c_{2}$ has emitter $E^{2}$. Each emitter $E^{i}$ evolves independently according to an emitter usage Markov chain, that is, whether the emitter is “on” or “off” is a dynamic process. This is actually the attribute evolution.

In addition, there are also measurement errors. That is, an object with class $c_{i}$ may be mistakenly declared by an ESM sensor, e.g., the state “on” may be declared by “off” or otherwise, the state “off” is declared by “on”. This is actually the attribute measurement model. More details about the attribute evolution and ESM measurement model can be found in [22].

## 3. EOT with Embedded Classification

Traditionally, for extended object tracking, only tracking is addressed without considering classification. This has many defects, since classification can usually assist tracking so as to achieve a better tracking performance. Furthermore, in some practical applications, classification is also required as a subproblem, i.e, we need to know the explicit object class label which can satisfy the practical demands for classification reality. More importantly, it can assist tracking to achieve a better performance.

Therefore, it is better to embed object classification into EOT, which can not only satisfy the practical demands for classification but also improve the tracking performance by utilizing the help of classification tracking. The knowledge of object class can help build more accurate motion models, which further assists tracking. However, in many traditional methods, this assistance is ignored because they may not realize that classification can assist EOT or they realize it but do not know how to utilize the classification information for improving tracking.

Considering this, we aim to handle this EOT problem with embedded classification by using all available data. In the following, we first introduce the object classification with multi-sensor data, which provides the basic knowledge about classification. Then, as the main part of this section, we propose a random-matrix-based multiple model EOT approach with soft classification and hard classification, respectively.

### 3.1. Classification with Multi-Sensor Data

The measurements at time *k* are denoted by $Z_{k}=\left\{Z_{k}^{x},Z_{k}^{c}\right\}$, where $Z_{k}^{x}$ and $Z_{k}^{c}$ are the kinematic measurements and attribute measurements, respectively. It is reasonable that the kinematic data and attribute data are independent given the object class, i.e.,:

$$\begin{array}{ccc} p(z_{k}^{x},z_{k}^{c}|x_{k},f_{k},c_{i},Z^{k-1}) & = & p(z_{k}^{x}|x_{k},f_{k},c_{i},Z^{k-1})\\ & & \times p(z_{k}^{c}|x_{k},f_{k},c_{i},Z^{k-1}) \end{array}$$

Then, according to the Bayesian rule, the probability of class $c_{i}$ is given as:

(4) $$P\left\{c_{i}|Z^{k}\right\}=\frac{1}{\delta }p\left\{Z_{k}^{x}|c_{i},Z_{x}^{k-1}\right\}P\left\{Z_{k}^{c}|c_{i},Z_{c}^{k-1}\right\}P\left\{c_{i}|Z^{k-1}\right\}$$

in which δ is the normalization factor, and $p\{Z_{k}^{x}|c_{i},Z_{x}^{k-1}\}$ and $P\{Z_{k}^{c}|c_{i},Z_{c}^{k-1}\}$ are the likelihoods of class $c_{i}$ based on the kinematic data and the ESM measurements, respectively. Here, i=1,⋯,N, where *N* is the maximum number of the object class. More details about the calculation of the likelihood $p\{Z_{k}^{x}|c_{i},Z_{x}^{k-1}\}$ and $P\{Z_{k}^{c}|c_{i},Z_{c}^{k-1}\}$ are given in [12] and [7], respectively.

### 3.2. Random-Matrix-Based EOT with Embedded Soft Classification

#### Random-Matrix-Based EOT

The purpose of the Bayesian estimator for model (1)–(3) is to obtain the conditional probability density function (pdf) $p[x_{k},X_{k}|Z^{k}]$ [12]:

$$\begin{array}{cc} p[x_{k},X_{k}|Z^{k}] & =p\left[x_{k}|X_{k},Z^{k}\right]p\left[X_{k}|Z^{k}\right]\\ \; & \propto N\left(x_{k};{\hat{x}}_{k},P_{k}⊗X_{k}\right)\mathrm{IW}\left(X_{k};{\hat{v}}_{k},{\hat{X}}_{k}\right) \end{array}$$

where ${\hat{x}}_{k}=E\left[x_{k}|Z^{k}\right]$, ${\hat{X}}_{k}={\bar{X}}_{k}\left({\hat{v}}_{k}-2d-1\right)$ with ${\bar{X}}_{k}=E\left[X_{k}|Z^{k}\right]$, MSE$\left({\hat{x}}_{k}\right)=\left(P_{k}⊗{\hat{X}}_{k}\right)/\left({\hat{v}}_{k}+b_{k}^{\mathrm{MSE}}\right)$, in which $b_{k}^{\mathrm{MSE}}=s-d-sd-3$ with *d* being the dimension of the space for the extended object and *s* being the dimension of the state in the one-dimensional of space, and IW is the inverted Wishart distribution. With the measurement $Z_{k}$, the Bayesian estimator updates $\left\{{\hat{x}}_{k-1},P_{k-1},{\hat{v}}_{k-1},{\hat{X}}_{k-1}\right\}$ to $\left\{{\hat{x}}_{k},P_{k},{\hat{v}}_{k},{\hat{X}}_{k}\right\}$.

For maneuvering EOT, a multiple model (MM) approach for state and extension estimation was derived in [12], which can significantly improve the overall tracking performance. Here:

$$p\left[x_{k},X_{k}|Z^{k}\right]=\sum_{l=1}^{M}p\left[x_{k},X_{k}|m_{k}^{l},Z^{k}\right]P\left\{m_{k}^{l}|Z^{k}\right\}$$

where $m_{k}^{l}$ denotes the event that model *l* is in effect at time *k*. *M* is the number of models in the MM approach. We adopt the following moment-matching-based multiple model EOT estimation strategy:

*State estimation:*

(5) $${\hat{x}}_{k}={\hat{x}}_{k}^{m}=\sum_{j}{\hat{x}}_{k}^{j}\mu_{k}^{j}$$

(6) $$P_{k}={\left[p_{i,j}\right]}^{s\times s}{\left({\left[p_{i,j}\right]}^{s\times s}\right)}^{T}$$

in which

$$\begin{array}{cc} p_{i,j} & =\frac{1}{d}\sum_{l=1}^{d}q_{(i-1)d+l,(j-1)d+l}\\ {}[q_{l,h}{]}^{sd\times sd} & ={\left[{\bar{v}}_{k}\left(I_{s}⊗{\hat{X}}_{k}^{-\frac{1}{2}}\right)P_{k}^{x,m}\left(I_{s}⊗{\hat{X}}_{k}^{-\frac{1}{2}}\right)\right]}^{\frac{1}{2}}\\ P_{k}^{x,m} & =\sum_{j}\mu_{k}^{j}\left[P_{k}^{x,j}+\left({\hat{x}}_{k}^{j}-{\hat{x}}_{k}^{m}\right){\left(\cdot \right)}^{T}\right]\\ P_{k}^{x,j} & =\left(P_{k}^{j}⊗{\hat{X}}_{k}^{j}\right)/\left({\hat{v}}_{k}^{j}+b_{k}^{MSE}\right)\\ {\bar{v}}_{k} & ={\hat{v}}_{k}+b_{k}^{MSE},b_{k}^{MSE}=s-d-sd-3 \end{array}$$

*Extension estimation:*

(7) $$\begin{array}{cc} {\hat{v}}_{k} & =\frac{1}{2}\left(A_{k}+\sqrt{A_{k}^{2}-8\left(B_{k}+2\right)}\right)+2d\\ {\hat{X}}_{k} & =\left({\hat{v}}_{k}-2d-2\right){\bar{X}}_{k}^{m} \end{array}$$

in which

$$\begin{array}{cc} {\bar{X}}_{k}^{m} & =\sum_{j}{\bar{X}}_{k}^{j}\mu_{k}^{j},{\bar{X}}_{k}^{j}={\hat{X}}_{k}^{j}/\left({\hat{v}}_{k}^{j}-2d-2\right)\\ P_{k}^{X,m} & =\sum_{j}\mu_{k}^{j}\left[P_{k}^{X,j}+\left({\bar{X}}_{k}^{j}-{\bar{X}}_{k}^{m}\right){\left(\cdot \right)}^{T}\right]\\ A_{k} & =B_{k}+C_{k}+5\\ B_{k} & ={\left[tr\left({\bar{X}}_{k}^{m}\right)\right]}^{2}/tr\left({\bar{X}}_{k}^{m}\right),C_{k}=\left[tr{\left({\bar{X}}_{k}^{m}\right)}^{2}\right]/tr\left({\bar{X}}_{k}^{m}\right) \end{array}$$

**Remark** **2.** *Based on the above estimation steps, we can obtain the state and extension estimate by (5) and (7), respectively. This random-matrix-based multiple model EOT approach is derived by moment matching, and the performance superiorities are fully demonstrated in [12]. Note that, in the above estimation steps, only kinematic data $Z_{x}^{k}$ are utilized.*

In Equation (5), the superscript *j* (j=1,⋯,M, where *M* is the number of models) means the *j*th model. In $p_{i,j}$ of Equation (6), i,j are only the indicative subscripts, which denotes the i,jth element in the s×s matrix ${\left[p_{i,j}\right]}^{s\times s}$.

Based on the state estimate (5), the extension estimate (7), and the object class probability (4), we propose the following EOT with an embedded soft classification strategy in Table 1.

The above algorithm provides both the tracking (containing the kinematic state estimate ${\overset{ˇ}{x}}_{k}$ and the extension estimate ${\overset{ˇ}{X}}_{k}$) and soft classification results. Note that in the tracking part, the involved class-dependent estimate ${\hat{x}}_{k}^{i}$ and ${\hat{X}}_{k}^{i}$ (here, the supersrcipt *i* denotes the *i*th object class, i=1,⋯,N) can be determined by (5) and (7), respectively. Essentially, the kinematic state estimate ${\overset{ˇ}{x}}_{k}$ is a weighted sum of all the hypotheses conditioned state estimate ${\hat{x}}_{k}^{i}$, where the weight is the corresponding class probability. Similarly, extension ${\overset{ˇ}{X}}_{k}$ is a weighted sum of all the hypotheses conditioned extension estimate ${\hat{X}}_{k}^{i}$.

For classification, there is no explicit class label, but only the class probability is provided. This is actually the so-called soft classification, which facilitates tracking by providing more accurate motion models. Furthermore, in this soft classification, the measurement $Z^{k}$ contains both the kinematic data $Z_{x}^{k}$ and the attribute data $Z_{c}^{k}$. With the assumption that $Z_{x}^{k}$ and $Z_{c}^{k}$ are conditionally (condition on target class), $Z^{k}$ can be given by (4).

**Remark** **3.** *It can be seen clearly that in this strategy, both the kinematic measurement (reflect in ${\hat{x}}_{k}^{i},{\hat{X}}_{k}^{i},P\left\{c_{i}|Z^{k}\right\}$) and the attribute measurement (reflect in $P\{c_{i}|Z^{k}\}$) are utilized in EOT tracking. Furthermore, with the assistance of attribute measurement, the correctness of class probability $P\{c_{i}|Z^{k}\}$ is improved, which finally results in better tracking performance.*

### 3.3. Random-Matrix-Based EOT with Embedded Hard Classification

In some practical EOT problems, the final goal is tracking, with explicit classification being an embedded subproblem; therefore, we should consider both tracking and a hard decision on object class. For a hard decision, the correctness and speed of decision making are the most important factors. In addition, considering that the measurements come sequentially, we propose the use of the well-known sequential probability ratio test (SPRT) for hard decisions for its nice properties. With SPRT, the type I and type II errors are controlled, and the expected sample size under both hypotheses are simultaneously minimized among all the tests. This fits the requirements of this paper well and is also simple for application. Therefore, we propose to use SPRT to obtain a hard decision.

Following SPRT, we first need to calculate the log-likelihood ratio (LLR):

(8) $$\begin{array}{cc} \; & L\left(Z^{k}\right)=\log P\left\{c_{2}|Z^{k}\right\}/P\left\{c_{1}|Z^{k}\right\} \end{array}$$

(9) $$\begin{array}{cc} \; & =\log\frac{p(Z_{k}|Z^{k-1},c_{2})}{p(Z_{k}|Z^{k-1},c_{1})}+\log\frac{P\{c_{2}|Z^{k-1}\}}{P\{c_{1}|Z^{k-1}\}} \end{array}$$

With the assumption that there is no prior information about the object class, the class-likelihood ratio is equivalent to the class posterior probability ratio when it is updated recursively. Then, the decision rule is given by:

(10) $$\begin{array}{c} L\left(Z^{k}\right)\leq \tau_{1}\\ \tau_{1}<L\left(Z^{k}\right)<\tau_{2}\\ L\left(Z^{k}\right)\geq \tau_{2} \end{array}\begin{array}{c} \mathrm{declare}\;c_{1}\\ \mathrm{soft}\;\mathrm{decision}\\ \mathrm{declare}\;c_{2} \end{array}$$

where the decision boundaries $\tau_{1}$ and $\tau_{2}$ are given as:

$$\tau_{1}=\log\frac{\beta }{1-\alpha },\;\tau_{2}=\log\frac{1-\beta }{\alpha }$$

where α,β are the type I error probability and type II error probability, respectively, which are constant over time. Note that the required probability of each class $P\{c_{i}|Z^{k}\}$ is provided in (4).

For the estimation part, we provide the following different strategies according to whether the hard decision on the class label is made [24]:

(a) Before the hard decision is made, the estimation is the same as that in EOT with embedded soft decisions.
(b) Once the hard decision is made, which means that the explicit target class is determined, we just need to perform tracking under the selected object class. For example, once decision on class $c_{i}$ is made, it is unchangeable, and thus, from this time on, the EOT estimation is (${\hat{x}}_{k}^{i},{\hat{X}}_{k}^{i}$), and the object class is always $c_{i}$. That is, the previous estimation which combines all class-dependent estimates are terminated, and only the estimation under class $c_{i}$ is output as the tracking result.

The above algorithm can be summarised as follows in Table 2:

**Remark** **4.** *Through the above EOT with embedded SPRT, we can obtain both tracking and classification results, as is desired. For classification, SPRT-based hard decision is the output, which enjoys the advantages of SPRT. For tracking, we provide different strategies according to the hard decision. Within this tracking strategy, the assistant of classification to tracking is fully reflected.*

**Remark** **5.** *For the hard decision, the decision bounds $\tau_{1}$ (the lower bound) and $\tau_{2}$ (the upper bound) are actually determined by the type I and type II errors (α and β), which are given parameters determined by practical problems. $\tau_{1}$ and $\tau_{2}$ are not time-varying since α and β are usually constant.*

The influence of $\tau_{1}$ and $\tau_{2}$ are as follows. The smaller $\tau_{1}$ is and the larger $\tau_{2}$ is (essentially, the smaller α and β are), the more difficult it is to make a hard decision, i.e., it requires longer steps to make a hard decision. On the contrary, the larger $\tau_{1}$ is and the smaller $\tau_{2}$ is (essentially, the larger α and β are), the easier it is to make a hard decision, i.e., it requires shorter steps.

### 3.4. Analyses of EOT with Embedded Classification

#### 3.4.1. Further Explanations of EOT with Embedded Classification

Generally, this paper proposes two strategies: EOT with embedded soft classification and EOT with embedded hard classification. They can not only satisfy the practical requirements but can also achieve superior tracking performance. These two strategies are applicable to different practical demands, and they also have their own advantages.

EOT with soft classification is applicable to the case that only tracking is required. In this case, the final result is tracking, and the embedded soft classification (class probability) is also provided to assist tracking. Essentially, correct classification can help improving tracking performance by providing more accurate class-dependent motion models. This assistant is fully utilized within the proposed EOT with embedded soft classification.

EOT with hard classification is applicable to the case where, in addition to the primary goal of tracking, an explicit hard decision on the target label is also required. Here, SPRT is adopted to obtain a hard classification result due to its nice properties, i.e, with controlled type I and type II errors, SPRT can make the quickest decision among all tests. In addition, according to whether the hard decision is made, different tracking strategies are creatively proposed.

More importantly, the proposed EOT approach with embedded (soft/hard) classification can utilize all available heterogeneous sensor data. That is because many attribute measurements cannot be used for tracking directly (since they do not contain target state information) but can be used for classification. In this case, through classification, these measurements can be utilized for tracking indirectly, which is beneficial for improving the tracking performance

**Remark** **6.** *As one of the main contribution, classification plays critical roles in the proposed EOT with embedded classification. First, in EOT with embedded soft classification, classification assists tracking since knowledge of target class information can help build more accurate motion models, which further benefits tracking. Second, in EOT with embedded hard classification, hard classification can satisfy the practical requirements on target class label. Third, through classification, the data which cannot originally be used for tracking directly (such data can only provide target identify information but cannot provide target state information) can be fully utilized for tracking. This further facilitates tracking.*

#### 3.4.2. Detail Analysis of the Contributions

The main contributions of the proposed EOT with embedded classification can be illustrated as follows.

Firstly, we explicitly formulates the extended object tracking problem with embedded classification while most existing literatures for extended objects only consider tracking.

Secondly, it is realized that although the final goal of many practical problems about extended object is tracking, class information is still incorporated, which may assist tracking. Essentially, correct classification can help improve tracking performance by providing more accurate class-dependent motion models. Therefore, we propose the EOT approach with embedded soft classification by fully utilizing the assistant of classification to tracking.

Thirdly, it is realized that, in practice, in addition to tracking, there is still another problem requiring an explicit hard decision on class label. Therefore, we propose the EOT approach with embedded hard classification. An SPRT-based hard decision is adopted due to its nice properties and its adaptability. Accordingly, appropriate tracking strategies are proposed.

Finally, both the proposed EOT with embedded soft and hard classification can utilize all available heterogeneous sensor data. However, many attribute measurements cannot be used for tracking directly (since they do not contain the object state information) but can be used for classification. In this case, through classification, these measurements can be utilized for tracking indirectly, which is beneficial for improving tracking performance.

## 4. Simulation and Discussion

In this section, two simple but representative extended object tracking problems with embedded classification are presented for illustration. Suppose there is only one extended target with two possible classes, which mainly differ in the maneuverability of the kinematic state and extension, as well as the attributes. Our ultimate and primary goal is to track the extended object and, meanwhile, handle the embedded classification subproblem according to the actual requirements.

Suppose there is only one extended object with two possible classes $c_{1}$ and $c_{2}$ described by the hybrid system (1)–(3). Note that total number of object classes must be known before the simulation experiment. In addition to the object state dynamics, $c_{1}$ and $c_{2}$ have different ESM attributes. For tracking, the multiple model method is adopted, and the model set for ω is {5π,7π,3π}/180 (rad/s) for class 1 and {−18π,16π,−16π,20π,−20π}/180 (rad/s) for class 2. Here, π=3.14. An equal prior probability for each model is assumed, and the transition probability matrix is omitted. The extended object is an ellipse with a long axis of 120 m and a short axis of 30 m. The assumed measurement noise (used for estimation) is distributed as $N(v_{k}^{r};0,\lambda X_{k}+R_{k})$ with λ=1/4 and $R_{k}=$ diag$\left[40^{2},40^{2}\right]$ m${}^{2}$. The number of measurements at each scan follows a Poisson distribution with a mean of 15. The sampling period is T=1 s. For each class *i*, $\delta_{k}^{i}=1$ and the initial extension $X_{0}^{i}=$ diag$\left[{\left(120/2\right)}^{2},{\left(30/2\right)}^{2}\right]$ m${}^{2}$. All results were obtained from 5000 MC runs, and the true target class is Bernoulli distributed with p=0.5. In SPRT, the controlled type I and type II error rates are α=β=0.01.

For the attribute feature, class $c_{i}$ has emitter $E^{1}$, and class $c_{2}$ has emitter $E^{2}$. The usage process for each emitter is as follows:

$$\Phi^{1}=\left[\begin{array}{cc} 0.7 & 0.3\\ 0.4 & 0.6 \end{array}\right],\Phi^{2}=\left[\begin{array}{cc} 0.8 & 0.2\\ 0.1 & 0.9 \end{array}\right]$$

The probability of “emitter on” at the initial time is assumed to be 0.5.

The ESM measurement process is assumed to be independent and given by:

$$P\left\{\mathrm{declare}\;E^{j}|\mathrm{true}\;E^{i}\right\}=\left\{\begin{array}{c} 0.8\;\;i=j\\ 0.2\;\;i\neq j \end{array}\right.i,j=1,2.$$

**Figure 1:**
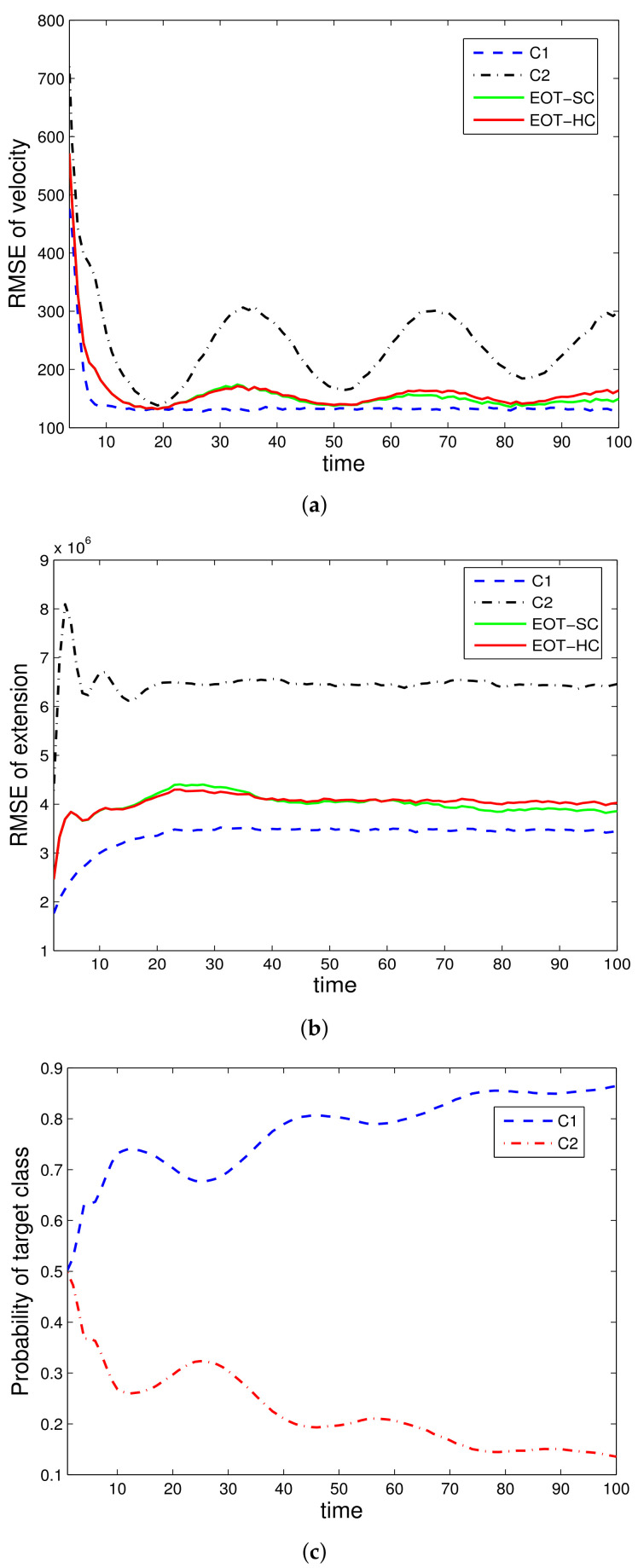
EOT-SC and EOT-HC using kinematic data, class *c*_1_ is true: (**a**) RMSE of velocity, (**b**) RMSE of extension, (**c**) Probability of target class.

**Figure 2:**
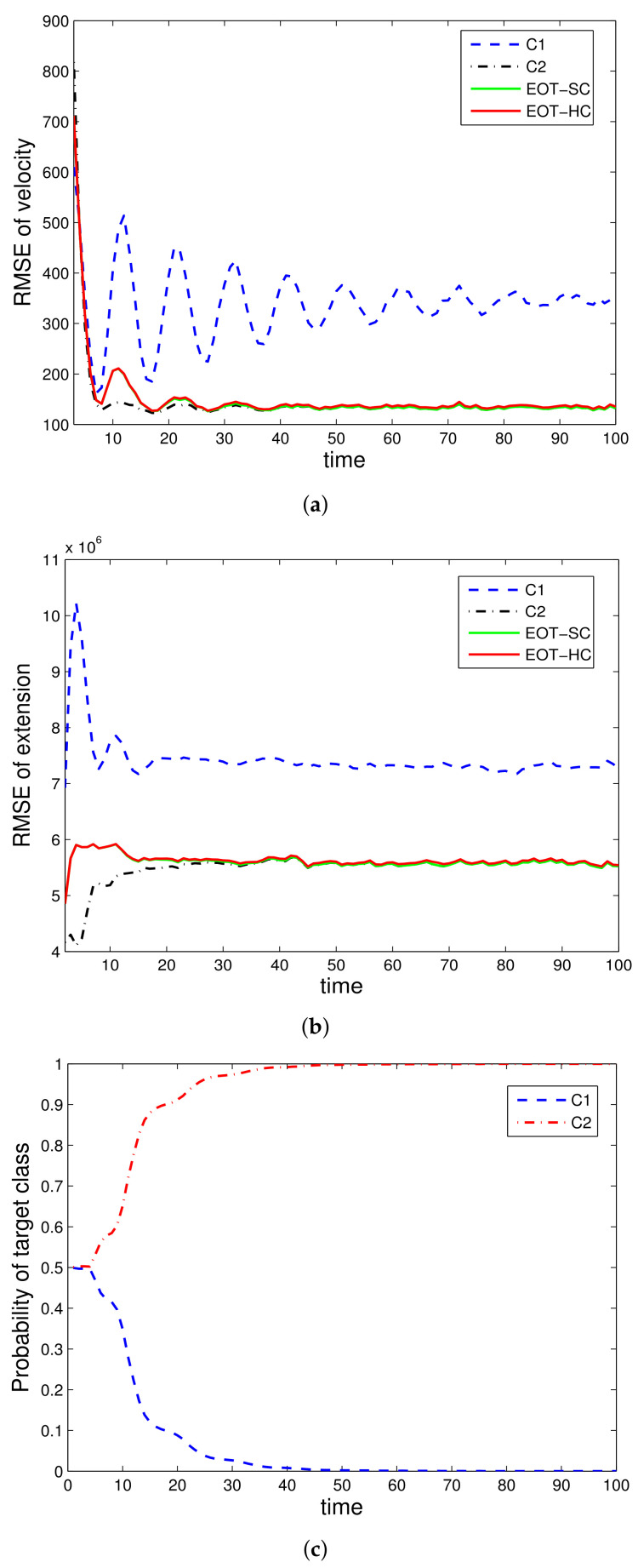
EOT-SC and EOT-HC using kinematic data, class *c*_2_ is true: (**a**) RMSE of velocity, (**b**) RMSE of extension, (**c**) Probability of target class.

**Figure 3:**
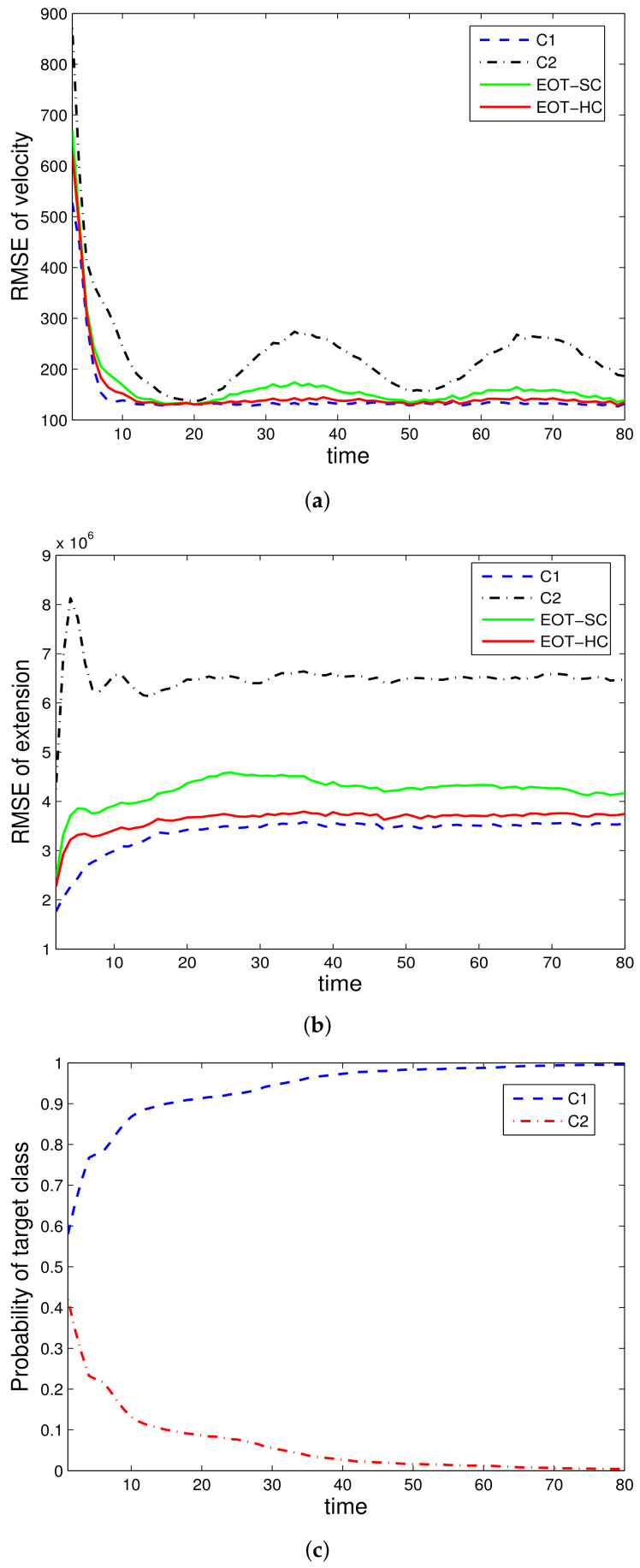
EOT-SC and EOT-HC using multisensor data, class *c*_1_ is true: (**a**) RMSE of velocity, (**b**) RMSE of extension, (**c**) Probability of target class.

**Figure 4:**
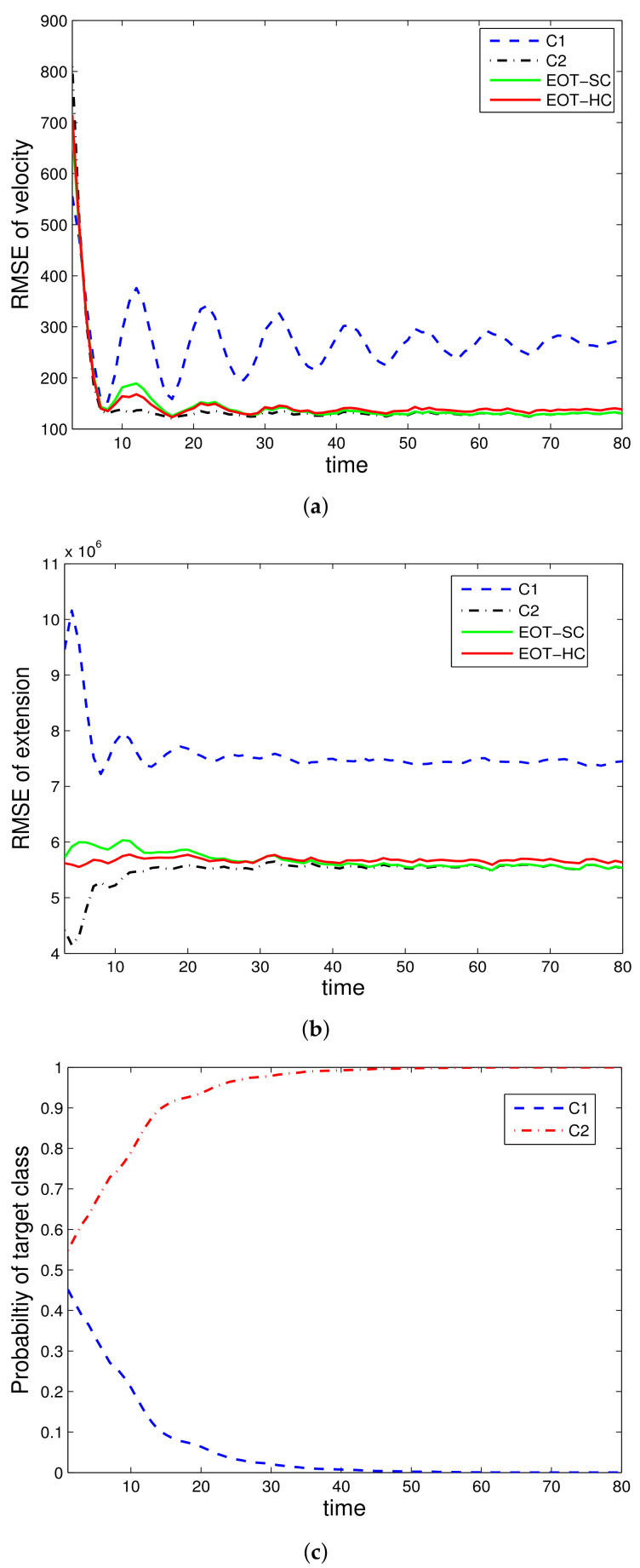
EOT-SC and EOT-HC using multisensor data, class *c*_2_ is true: (**a**) RMSE of velocity, (**b**) RMSE of extension, (**c**) Probability of target class.

Two illustrative simulation examples are presented. In simulation 1, only kinematic data are utilized, with the performance results shown in Figure 1 and Figure 2. In simulation 2, both kinematic and attribute data are utilized, with the results shown in Figure 3 and Figure 4. To make it clear, we priovide the following more detailed explanations of the figures:

Figure 1 is the simulation results with only kinematic measurement under the true class $c_{1}$; Figure 2 is the simulation results with only kinematic measurement under the true class $c_{2}$; Figure 3 is the simulation results with both kinematic and attribute measurements under the true class $c_{1}$; Figure 4 is the simulation results with both kinematic and attribute measurements under the true class $c_{2}$.

The evaluated performances are velocity estimation RMSE, extension estimation RMSE, and estimated class probability of each target class. For denotation simplicity, “EOT-SC” and “EOT-HC” mean EOT with embedded soft classification and EOT with embedded hard classification, respectively. “C1” and “C2” mean the corresponding performance under class $c_{1}$ and class $c_{2}$, respectively. In each simulation run, the true target class remains constant throughout the whole time steps.

Figure 1 and Figure 2 verify the effectiveness of the proposed EOT-SC and EOT-HC. The tracking errors of both EOT-SC and EOT-HC are between those of using classes $c_{1}$ and $c_{2}$, and as time goes on, their performances become close to that under the true target class. This verify that with classification-aided tracking (EOT-SC and EOT-HC both belong to this category), the tracking performance converges to that under the true category soon.

For classification performance, since EOT-SC and EOT-HC involve soft classification and hard classification, respectively, we analyze them separately. Figure 1c and Figure 2c are the soft classification result under class $c_{1}$ and class $c_{2}$, respectively. It can be seen clearly that with the accumulation of data, the probability of the true class converges to 1; meanwhile, the probability of the opposite class converges to 0. This demonstrates the effectiveness of the soft classification. For hard classification, with SPRT, it is guaranteed that the actual error rate is no greater than the controlled type I and type II errors.

Figure 3 and Figure 4 show the tracking performance of the proposed EOT-SC and EOT-HC using both the kinematic and attribute data. The law is basically consistent with Figure 1 and Figure 2, where both EOT-SC and EOT-HC have superior tracking performance. However, there are still differences. Generally, with the help of attribute measurements, both the tracking and classification performances are improved.

Specifically, comparing Figure 1 with Figure 3 (they are both under class $c_{1}$), there are two main differences. For classification, it can be seen clearly that with attribute measurement, the soft classification performance is greatly improved. That is, Figure 3c greatly outperforms Figure 1c. For tracking, with the introduction of attribute measurements, both the velocity and extension estimation RMSE are lowered. That is, Figure 3a,b outperform Figure 1a,b. Comparing Figure 2 with Figure 4 (they are both under class $c_{2}$), the differences follow the same law with the difference between Figure 1 and Figure 3.

However, the differences between Figure 2 and Figure 4 are much smaller than the differences between Figure 1 and Figure 3. That is because it is easier to distinguish different target classes when $c_{2}$ is true than that when $c_{1}$ is true. Therefore, the help of attribute data in this “easy” case is not as obvious as its help in the “difficult” case. It actually reflects the superiority of the proposed method that the more difficult the scenario is, the more obvious the advantage is.

**Remark** **7.** *Generally, the comparisons of the results indicate that with the help of attribute data, the tracking performance of EOT is greatly improved. More specifically, the classification performance is improved due to the introduction of attribute data, which contain the target identity information. Then, the tracking performance is further improved due to the improvement of the classification performance. This assistant effect is more obvious when the classes are difficult to be distinguished.*

**Remark** **8.** *The proposed EOT-SC and EOT-HC methods can meet the practical requirements of EOT with embedded classification. We can choose either EOT-SC or EOT-HC according to the practical demands, i.e., whether an explicit hard decision on class label is required. Generally, with both methods, we can obtain superior tracking performance, which is our most important goal.*

## 5. Conclusions

This paper proposes a novel, random-matrix-based, extended object tracking (EOT) approach with embedded classification. The traditional method addressing extended objects mainly focuses on object tracking, which not only cannot meet the actual needs (sometimes classification is needed) but also leads to limited tracking performance (class information may assist tracking but is usually ignored). Therefore, this paper explores appropriate EOT methods with embedded classification.

Firstly, we formulate the EOT problem with embedded classification by appropriate modeling, in which both the kinematic and attribute data are taken into account. Then, two strategies are proposed, oriented to actual demands, which are the EOTs with soft classification (output the class probability only) and hard classification (output the explicit class label), respectively. Specifically, for tracking, the random-matrix-based EOT method with a multiple model approach is adopted; for classification, all available data are utilized for improving the classification performance (which further assists target tracking). Furthermore, for EOT with embedded hard decision, SPRT is adopted due to its nice properties and adaptability to our problem.

Our simulation results verify the effectiveness of the proposed EOT with an embedded classification approach. It can output the required tracking and classification result. Furthermore, by introducing more heterogeneous data and the assistance of classification, the final tracking performance is improved. In the future, multiple extended object tracking with more types of multi-sensor data would be investigated.

## Figures and Tables

**Table 1:** EOT algorithm with embedded soft classification.

**Tracking**	$\begin{array}{c} \mathrm{kinematic}\;\mathrm{state}:\;{\overset{ˇ}{x}}_{k}=\sum_{i}{\hat{x}}_{k}^{i}P\left\{c_{i}|Z^{k}\right\}\\ \mathrm{Extension}:\;\;\;\;\;{\overset{ˇ}{X}}_{k}=\sum_{i}{\hat{X}}_{k}^{i}P\left\{c_{i}|Z^{k}\right\} \end{array}$
**Classification**	$P\left\{c_{i}|Z^{k}\right\}=\frac{1}{\delta }p\left\{Z_{k}|c_{i},Z^{k-1}\right\}P\left\{c_{i}|Z^{k-1}\right\}$

**Table 2:** EOT algorithm with embedded hard classification.

**Tracking**	$\begin{array}{c} \mathrm{Before}\;\mathrm{decision}\;\mathrm{is}\;\mathrm{made},\;\left\{\begin{array}{c} {\overset{ˇ}{x}}_{k}=\sum_{i}{\hat{x}}_{k}^{i}P\left\{c_{i}|Z^{k}\right\}\\ {\overset{ˇ}{X}}_{k}=\sum_{i}{\hat{X}}_{k}^{i}P\left\{c_{i}|Z^{k}\right\} \end{array}\right.\\ \mathrm{After}\;\mathrm{decision}\;\mathrm{on}\;\mathrm{class}\;c_{i}\;\mathrm{is}\;\mathrm{made},\;\left\{\begin{array}{c} {\overset{ˇ}{x}}_{k}={\hat{x}}_{k}^{i}\\ {\overset{ˇ}{X}}_{k}={\hat{X}}_{k}^{i} \end{array}\right. \end{array}$
**Classification**	$\left\{\begin{array}{c} \mathrm{Decide}\;\mathrm{on}\;c_{1}\;\mathrm{if}\;L\left(Z^{k}\right)\geq \tau_{1}\\ \mathrm{No}\;\mathrm{hard}\;\mathrm{decision}\;\mathrm{is}\;\mathrm{made},\;\mathrm{continue}\\ \mathrm{Decide}\;\mathrm{on}\;c_{2}\;\mathrm{if}\;L\left(Z^{k}\right)\leq \tau_{1} \end{array}\right.$

## Data Availability

Not applicable.
